# Effects of the Central Unit Structure, Lateral Substitution and Symmetry on the Mesomorphic Behavior of Some Bent‐Core Azoester Derivatives

**DOI:** 10.1002/open.202400454

**Published:** 2025-02-21

**Authors:** Catalina‐Ionica Ciobanu, Elena‐Luiza Epure, Laurentiu Soroaga, Aurel Simion, Gabriela Lisa, Irina Carlescu

**Affiliations:** ^1^ Department Institute of Interdisciplinary Research-CERNESIM Centre Institution Alexandru Ioan Cuza University of Iasi 11 Carol I Iasi 700506 Romania; ^2^ Department ”Cristofor Simionescu” Faculty of Chemical Engineering and Environmental Protection Institution Gheorghe Asachi Technical University of Iasi 73 Prof.dr.doc. D. Mangeron Street 700050 Iasi Romania; ^3^ RECENT-AIR Centre Alexandru Ioan Cuza University of Iasi 11 Carol I Iasi 700506 Romania

**Keywords:** Bent-core molecules, Thermal properties, Mesophase, Liquid crystals, Molecular modeling

## Abstract

We report here the synthesis and characterization of some new bent‐core asymmetric compounds derived from resorcinol whose thermal behavior has been analyzed by comparison with their analogs derived from 1,3‐disubstituted benzene and 2,7‐dihydroxynaphthalene containing azoester aromatic units and alkyl chain end. The asymmetric structures contain 4‐(4‐alkyloxyphenylazo)‐benzoyl and 4‐methoxy‐benzoyl or 3‐bromo‐4‐methoxy‐benzoyl as side arms. The investigations have been carried out to reach a better understanding of the structure‐properties relationship in such bent‐shaped compounds. We observed that a change in molecular structure like the nature of the central core, the symmetry of the structure or the presence of polar lateral substituent influence not only liquid crystalline properties, but also the thermal behavior. The thermogravimetric analysis showed that the bent‐core derivatives have a good thermal stability since the degradation of the compounds begins over the isotropization temperature. Theoretical calculations were performed to elucidate the behavior of the compounds. These can assist us in designing new molecules that exhibit specific mesomorphic properties.

## Graphical abstract

The thermal behavior of some bent‐core analogous derivatives was investigated. Theoretical calculations using molecular dynamics were performed, to elucidate the different mesomorphic behavior of compounds. The crystal structure was minimized at the quantum level, then introduced into a cell that multiplied along the three directions of the orthogonal space. The obtained lattice of 27 molecules was further energetically optimized.

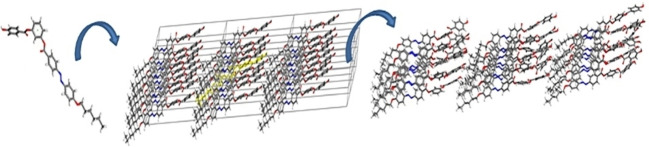

## Introduction

Liquid crystalline materials are versatile materials due to their unique electrical, optical and mechanical properties, where a minor change in structure significantly changes the phase behavior.[^1^, ^2^, ^3^] The properties induced by the azo linkage into molecules make bent‐core compounds valuable in the field of materials science. The main characteristic of such compounds is the photosensitivity of the materials, which are capable of structural changes upon irradiation with light that lead to modification of their optical properties and thus contribute to device applications.[^4^, ^5^, ^6^, ^7^, ^8^, ^9^] The appearance of liquid crystalline properties depends on structural parameters like rigidity or flexibility but is influenced as well by alterations in polarity and polarizability due to changes in terminal polar substituents.^[10]^ These materials have been directed towards display applications, semiconductors or optical devices. It is also known that mesomorphic behavior of such compounds is influenced by linking groups or lateral substituents.[^11^, ^12^] Thus, the addition of a lateral unit to a mesogenic structure enhances the molecular size and decreases the lateral interactions of rod‐shaped molecules with decreasing of melting point, as well as mesophase stability.[^13^, ^14^]

The bending angle and the terminal or lateral substitutions have a major impact on the mesomorphic behavior and physical properties.[^15^, ^16^] Additionally, the stability of the formed mesophases and their behavior are largely influenced by key factors such as the polarity of the substituents, polarizability, molecular flexibility, and overall design. It is known that good polarizability of the mesogenic core of the molecule increases the stability of a mesophase.^[17]^ Also, the connecting groups between rigid structures, such as phenyl rings, help those compounds to maintain the structural linearity and influence the mesophase stability.^[18]^

Banana‐shaped compounds contain a central core and two side arms connected in a such way to ensure the favorable bending angle. Typically, 1,3‐benzene or 2,7‐naphthalene derivatives are the most used disubstituted central cores, considering the bending angle of about 120° favors stable mesophases. Resorcinol was the first used to obtain bent‐core compounds with liquid crystalline properties with smectic or nematic phases.[^19^, ^20^, ^21^]

Moreover, incorporating the azobenzene moiety into bent‐core derivatives is beneficial for obtaining new materials, because induces changes in physical properties, maintains the molecules’ linearity and stiffness and helps to maintain a stable mesophase.[^22^, ^23^, ^24^] On the other hand, the π‐electron associated with the presence of polar carbonyl group of the ester linking group gives promising functionality towards thermal stability, ease of synthesis, and lower melting points.[^25^, ^26^]

**Figure 1:**
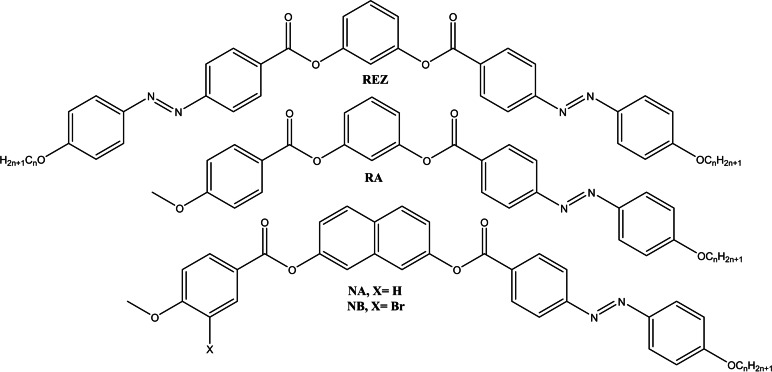
General structures of bent‐core azo‐ester derivatives (n=6–10).

Here we have synthesized and characterized a series of new asymmetric bent‐core compounds RA with terminal flexible alkylated chains with six to ten carbon atoms. Their thermal behavior was discussed by comparation with analogous derivatives (Figure 1) based on 2,7‐dihydroxynaphtalene (NA and NB) and resorcinol (REZ) as central units synthetized in our group and reported earlier.[^26^, ^27^]

The main aspects focused on thermal characterization, surface texture and theoretical calculations, considering the structural modifications due to central units or addition of lateral substituent to the side arm.

## Results and Discussion

### Synthesis

**Scheme 1:**
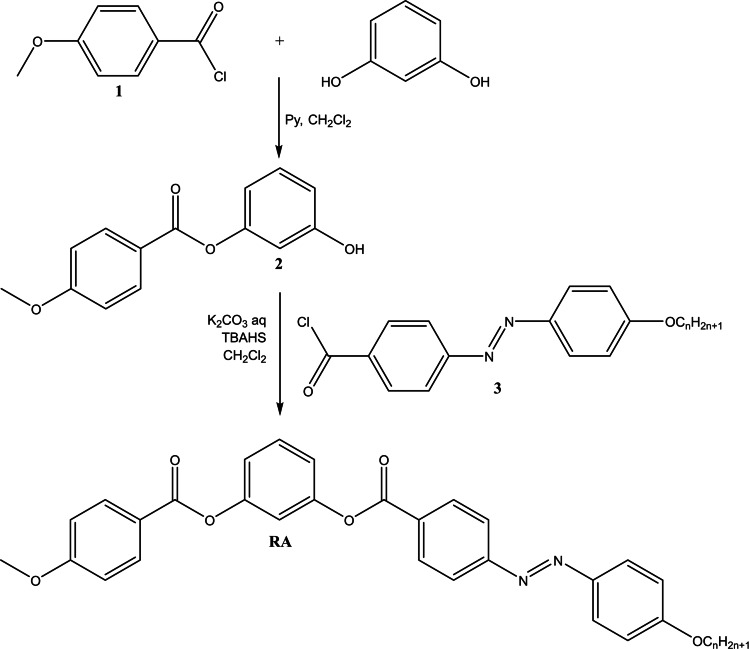
Synthesis of asymmetric bent‐core compound (RA, n=6–10).

The compounds RA6–10 derived from resorcinol were prepared in a convergent manner (Scheme 1). First, resorcinol was reacted with 4‐methoxy‐benzoyl chloride (1) in presence of pyridine, in anhydrous dichloromethane, to afford the monoester 1‐(4‐methoxyphenylcarbonyloxy)‐3‐hydroxybenzene (2).^[28]^ Next, the monoester (2) was reacted with 4‐(4‐alkyloxyphenylazo)‐benzoyl chlorides (3) in aqueous K_2_CO_3_/dichloromethane and tetrabutylammonium hydrogen sulfate (TBAHS). After stirring for 48 h at room temperature, the organic phase was separated, washed two times with water, dried over MgSO_4_ and evaporated. The resulted compounds were purified by column chromatography (Silicagel/methylene chloride:ethyl acetate=20 : 1).

### Mesomorphic Properties

**Figure 2:**
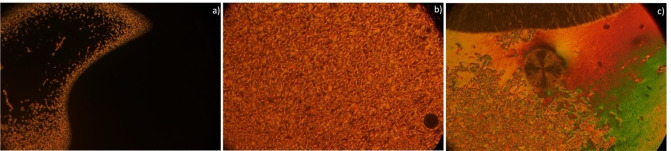
Optical photomicrographs textures of NA7. a) The isotropic phase transforming into LC2 phase at 130 °C in homeotropic state with characteristic Schlieren nematic texture, b) The LC1 phase upon cooling to 123 °C, c) Spherulite growing from nematic phase at 104 °C.

Optical microscopy analysis under polarized light revealed no liquid crystalline properties for any of RA compounds (Table 1). They simply melt at temperatures between around 103 °C and 109 °C, much lower compared with their analogs. In terms of NA and NB analogs, the changing of benzene into naphthalene central core favored the mesomorphic properties evidenced by nematic phase with characteristic Schlieren texture (Figure 2) but only on cooling, except NB10 compound that is the most stable in terms of mesophases.

**Table 1:** Phase transition temperatures (°C) and Transition enthalpies [ΔH, J/g] determined by DSC (scan rate 10 °C/min).

Comp	n	Phase transitions	Comp	n	Phase transitions
RA6	6	Cr 109* Iso	NA6	6	Heating: Cr 140 [48.72] Iso Cooling: Iso 137* LC_2_ 130 [0.24] LC_1_ 96 [46.29] Cr
RA7	7	Cr 103* Iso	NA7	7	Heating: Cr 133 [36.87] Iso Cooling: Iso 130* LC_2_ 124 [0.22] LC_1_ 104 [42.56] Cr
RA8	8	Cr 104* Iso	NA8	8	Heating: Cr 140 [47.85] Iso Cooling: Iso 135* LC_2_ 127 [0.2] Lc_1_ 108 [29.37] Cr
RA9	9	Cr 105* Iso	NA9	9	Heating: Cr 150 [59.04] Iso Cooling: Iso 146* LC_2_ 131 [8.47] LC_1_ 107 [2.81] Cr
RA10	10	Cr 106* Iso	NA10	10	Heating: Cr 146 [55.36] Iso Cooling: Iso 142* LC_2_ 112 [0.21] LC_1_ 92 [39.33] Cr
REZ6	6	Heating: Cr_1_ 116 [0.42] Cr_2_ 168 [40.81] Iso Cooling: Iso 163 [15.54] LC 153 [16.81] Cr_2_ 113 [0.71] Cr_1_	NB6	6	Heating: Cr 149 [48.27] Iso Cooling: Iso 99* LC 86 [21.85] Cr
REZ7	7	Heating: Cr_1_ 111 [30.38] Cr_2_ 142* LC 163 [66.55] Iso Cooling: Iso 153* LC 137 [29.17] Cr_2_ 115 [10.7] Cr_1_	NB7	7	Heating: Cr 159 [45.83] Iso Cooling: Iso 108* LC 103 [19.32] Cr
REZ8	8	Heating: Cr_1_ 94 [6.7] Cr_2_ 143* LC 159 [64.96] Iso Cooling: Iso 155* LC 147 [49.80] Cr_2_ 119 [10.07] Cr	NB8	8	Heating: Cr 147 [45.25] Iso Cooling: Iso 109* LC 98 [27.35] Cr
REZ9	9	Heating: Cr_1_ 100 [0.18] Cr_2_ 147 [63.84] Iso Cooling: Iso 133 [48.58] Cr_2_ 111 [17.08] Cr_1_	NB9	9	Heating: Cr 129 [32.03] Iso Cooling: Iso 106 [0.35] LC 91 [11.72] Cr
REZ10	10	Heating: Cr_1_ 100 [3.83] Cr_2_ 148 [63.84] Iso Cooling: Iso 130 [38.62] Cr_2_ 109 [0.81] Cr_1_	NB10	10	Heating: Cr 130 [0.28] LC 146 [0.28] Iso Cooling: Iso 135 [0.94] LC 107 [32.51] Cr

* data obtaining from POM.

The symmetrical REZ compounds derived from resorcinol exhibit rich polymorphism due to their bulkier structures, which order into a mesophase. Thus, only the middle derivatives REZ7 and REZ8 present mesophases on heating and cooling, while the first compound REZ6 display mesophase only on cooling and the last two compound REZ9 and REZ10 did not show liquid crystalline properties.^[29]^

**Figure 3:**
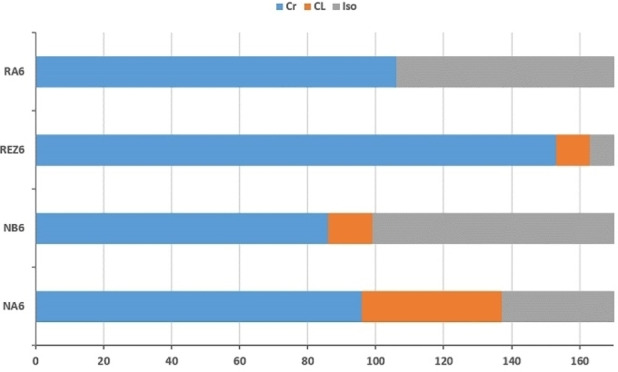
Temperature transitions on cooling for bent‐core derivatives.

Considering this, next we performed a comparative study only for the first compounds in each series, to follow the mode of variation of mesomorphism (Figure 3). One can say that the mesophase temperature transitions were influenced both by the size of the central core and the position of the substituent on calamitic arm.

For example, compound NA6 exhibits the widest mesophase domain compared with compound with bromine lateral substituent NB6, seeming the presence of the bulky atom influences self‐assembling of molecules into stable phase. On the other side, the introduction of second arm into symmetric resorcinol bent‐core derivative REZ6 induce the liquid crystalline properties but on narrower range, compared with NA6, with increasing the isotropisation temperature.

### Thermal Analysis

**Figure 4:**
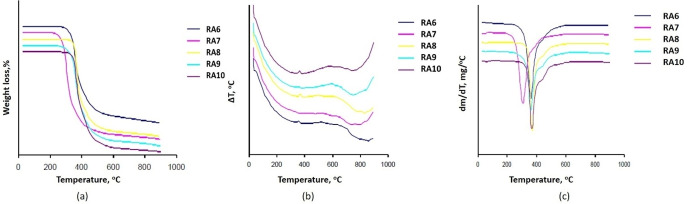
TG (a), DTA (b) and DTG (c) curves for bent‐core asymmetric compounds RA6–10.

Thermogravimetric study of the compounds RA6–10 indicates a trend of odd‐even effect, considering T_onset_ temperature of the degradation step (Figure 4). It was observed that derivatives with an even number of carbon atoms in the terminal chain (6, 8, 10) behave a higher thermostability than those with odd number of carbon atoms in the terminal chain (7, 9).

The TGA analysis showed that the thermal stability of the analogous compounds is relatively high since the degradation process begins over 250 °C, much higher than the isotropization temperature (Table 2). All the derivatives present a single stage of degradation with significant mass losses, except compund REZ6 that decomposes thermally in two steps with less mass loss than other compounds.

**Table 2:** Thermal data for bent‐core azoester derivatives.

Comp.	Ti°C	1st decomp. temp (°C)	wt.loss (%)	2nd decomp. temp (°C)	wt.loss (%)
NA6	140	349–591	65.97	–	–
NB6	149	256–559	67.69	–	–
REZ6	168	356–371	30.73	398–578	38.52
RA6	109	316–537	76.60	–	–

Ti – isotropization temperature.

### SEM Analysis

**Figure 5:**
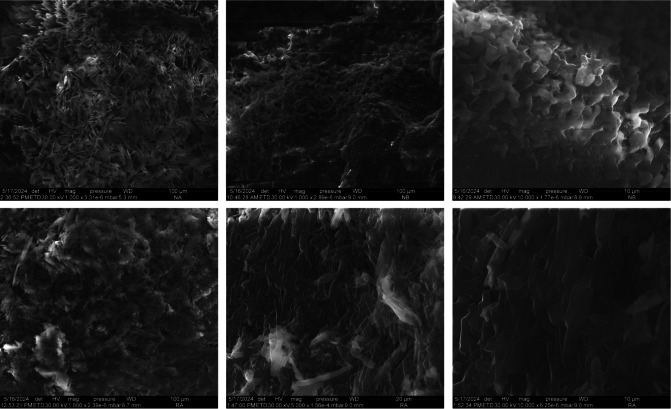
Scanning electron microscopy. SEM images of NA6, NB6 (up) and RA6 (down).

Following the comparative study for the first compound in each series and considering non mesomorphic character of the RA series, SEM analysis was performed (Figure 5) without any mechanical sample processing in order to evidence the polymorphism and crystalline state of such systems.

For 2,7‐dihydroxynaphthalene bent‐core derivatives NA6 and NB6, the bulk sample morphologies observed at SEM analysis show an apparent roughness in the fracture surfaces at a high‐resolution magnified image of 100 μm. On the other hand, the 10 μm enlarged image for the NB6 sample reveals a well‐ordered structure. Layered sheet‐like pieces were discovered in the instance of the resorcinol asymmetric derivative RA6.

### Molecular Modeling

Motivated by the experimental data, indicating that RA6 compounds do not exhibit liquid crystalline properties, compared with their analogs, theoretical calculations were also performed. The energies of the structures with different degrees of ordering were determined using molecular dynamics. In this regard, two structure sets were built: Set I, which imitates a crystalline structure, and Set II, with nematic ordering. More construction details can be found in Materials and Methods.

### Preparation and Characterization of Set I of Compounds

**Figure 6:**
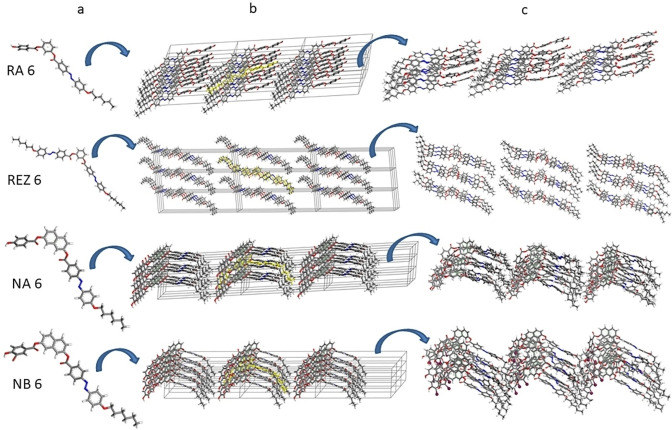
Schematic diagram of the sistem I construction: (a) minimized isolated molecules, (b) crystal lattice (the molecule in the main cell is marked in yellow), (c) crystalline structure (in the NB6 structure, the bromine atoms are represented by a grainy ball).

To observe the transition of compounds from the crystalline to the disordered phase, lattices were first constructed to simulate the crystalline state. The crystal structures^[30]^ were constructed as follows: (1) the structures were minimized at the quantum level (Figure 6a) and the length and width of the molecules were measured (data are given in Table 3), (2) a single molecule was introduced into a cell having the sizes corresponding to those in Table 4, (3) the cell was multiplied along the three directions of the orthogonal space, obtaining a lattice of 27 molecules (Figure 6b), (4) the structures were energetically optimized (Figure 6c). Then, Set I was subjected to molecular dynamics simulations for 500 ns at room temperature (T=298 K) and temperature T1 (as detailed in Materials and Methods).

**Table 3:** Structural details of energy‐optimized molecules.

Molecule	RA6	REZ6	NA6	NB6
lenght	30.4	43.6	31.1	31.1
width	11.5	9.9	8.9	9.6

**Table 4:** Values of the spatial orientation correlation function, P2(r), for the ordered systems, set II.

System	RA6	REZ6	NA6	NB6
P2(r)	0.35	0.61	0.72	0.68

The energy difference between the disordered state at temperature T1 and the crystalline state was calculated as: Δ E=E (T1)−E (298 K), where E (T1) and E (298 K) represent the energies for the set I at the temperature corresponding to mesophase and room temperature, respectively.

The results were as follows: Δ E (RA6)=−73.5 kcal/mol, Δ E (REZ6)=−7.1 kcal/mol, Δ E (NA6)=71.2 kcal/mol, Δ E (NB6)=58.4 kcal/mol. Theoretical data indicate that the RA6 structure prefers a disordered state, unlike the NA6 and NB6 compounds. The positive energy differences of NA6 and NB6 suggest that disordered structures are not characteristic of these compounds at the T1 temperature. It is important to note that RA6 does not exhibit liquid crystalline properties, while NA6 and NB6 compounds display nematic structures. The slightly negative value, close to zero, of REZ6 could be controversial, considering that smectic phases are more ordered than nematic ones. However, the value for REZ6 is somewhat positioned in the middle of the range defined by the extreme values, suggesting a different behavior for REZ6 compared to the other compounds.

### Preparation and Characterization of Set II of Compounds

**Figure 7:**
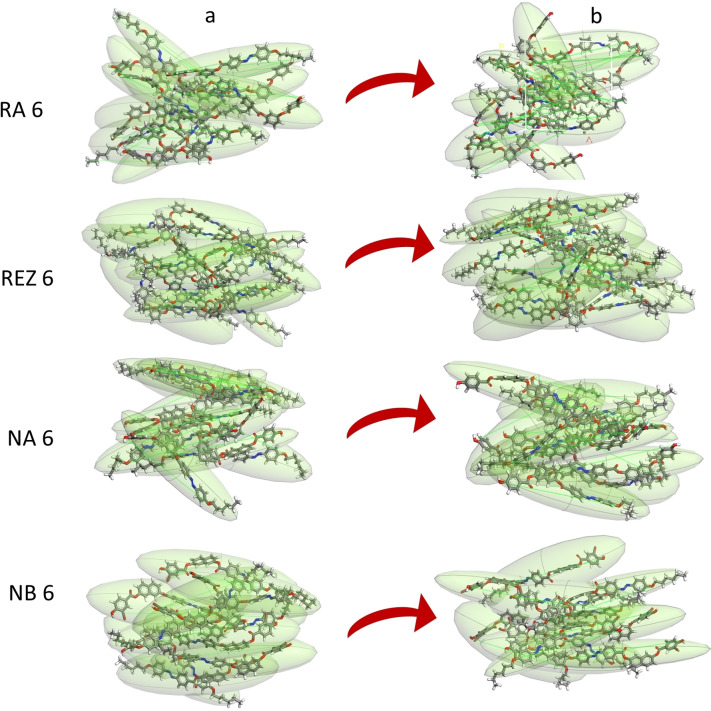
Images of the structures in the starting set II (a) and at mesophase temperature (b). Molecules have been included in ellipses for better visualization of their ordering.

Another set of structures, II, was constructed by including 10 molecules in an amorphous cell with nematic ordering (construction details are in Materials and Methods). The geometrically optimized structures were subjected to a protocol involving anneal dynamics in NVT (5 cycles, 300–700 K) and NPT (500 ps) assemblies. Optimization of the structures was done after each cycle. The production run was performed at the T1 temperature. The plots of temperatures and densities showed that they reached a plateau value after 10 ps. In Figure 7 the starting and final cells for each compound are plotted.

To quantitatively express the degree of ordering within the Set II structures, the spatial orientation correlation function, P2(r), was calculated and the data are given in Table 4.

So, the theoretical data also confirm that the RA6 structure cannot form an ordered mesophase. Compounds NA6 and NB6 exhibit the highest values of the P2(r) function, confirming the nematic structures experimentally observed. For Set I, NB6 shows a slightly lower degree of order compared to NA6, due to the presence of the bulky bromine atom. It was expected that the value of the spatial orientation correlation function for REZ6 would be higher, considering that, in reality the mesophase is a nematic one. These results suggest, once again, a different behavior of REZ6 compared to the analyzed compounds.

## Conclusions

Novel molecules have been successfully synthesized, and their mesomorphic properties were investigated. We observed that the liquid crystalline properties of compounds can be altered by structural changes such as the type of central core, the overall length of the molecular structure, the lateral substitution on side arms and length of lateral arms. All the synthetized asymmetric bent‐core compounds derived from resorcinol (RA6) are non mesomorphic while symmetric analogous (REZ6) are enantiotropic or monotropic. Changing the central unit from benzene into naphthalene while maintaining the asymmetry of structures induce mesomorphism but analogs compounds (NA6 and NB6) are only monotropic. The structural morphologies were evidenced by SEM.

We simulated systems with different degrees of ordering of molecules. Each time it was confirmed that the RA6 structure does not resist to mesophases. Despite RA6 lacking liquid crystalline properties, we proceeded to create compounds with extended side chains to see if this would influence their mesomorphic behavior. It was observed that a small central core lead to less orderly packed molecules. The presence of nematic phase was highlighted for the NA6 and NB6 structures, due to their increased length‐to‐width ratio and higher longitudinal dipole moment of the compounds along the long molecular axis. These findings could aid in exploring new opportunities to design and develop the newly molecules usefulness in functional devices.

## Experimental Section

### Materials and Methods

The synthesis of the compounds derived from 2,7‐dihydroxynaphtalene, (NA6 and NB6) and the symmetrical compound derived from 1,3‐resorcinol, (REZ6), were previously reported.[^27^, ^29^] 3‐Bromo‐4‐methoxy‐benzoic acid, 4‐methoxy‐benzoyl chloride, 3‐bromo‐4‐methoxy‐benzoyl chloride and 4‐(4‐hexyloxyphenylazo)‐benzoyl chloride used in intermediary reactions were prepared according to literature data.[^31^, ^32^] All other materials used in the present work were purchased from Sigma Aldrich and used as received. For column chromatography Silica gel 60 (Merck) was used, while for thin layer chromatography Silica gel plates (Merck Silica gel F254).

Structural characterization by ^1^H‐NMR and ^13^C‐NMR of RA6 compound was made using a Bruker Avance III, 500 MHz spectrometer (Germany) equipped with a 5 mm PABBO detection probe and operating at 500.19 MHz for 1H, and 125.78 MHz, respectively, for 13 C nuclei. For NA6 and NB6 compounds a Bruker Avance DRX 400 MHz NMR spectrometer was used for the spectra recorded^[27]^ Chemical shifts are in δ unit (ppm) with the residual solvent peak as the internal standard. The coupling constant (J) is reported in Hertz (Hz). The following abbreviations were used to designate chemical shift multiplicities: s singlet, d doublet, t triplet, m multiplet, qv quintet. Mass spectra for RA6 compound were recorded using a mass spectrometer Agilent 6520 Accurate Mass Q‐TOF LC=MS (Santa Clara, CA, USA) equipped with an electrospray ion source.

The compounds’ textures were examined under polarized light with a cross polarizer, where the sample was prepared as a thin film between a glass slide and coverslip. It was used an Axioscop 40 Zeiss polarizing optical microscope equipped with a Linksys 32 hotstage temperature control unit (Linkam Scientific Instruments Ltd., Tadworth, UK). Mettler Toledo DSC equipment was used to highlight the phase transitions occurring in the analyzed materials, which allowed the recording of DSC curves in an inert atmosphere with a heating rate of 10 °C/min and a sample mass between 2.4 and 5.0 mg. Three heating steps and two cooling steps were performed in the temperature range of 25–300 °C.

The evaluation of the thermal stability of the synthesized materials was carried out by thermal gravimetric analysis (TGA), using Mettler Toledo 851^e^ equipment (Mettler Toledo, Greifensee, Switzerland) in an inert atmosphere with a nitrogen flow rate of 20 mL/min and a heating rate of 10 °C/min from 25 °C to 900 °C. The mass of the samples subjected to thermogravimetric analysis ranged from 2.5 to 4.7 mg.

The images have been recorded using the Quanta 250 (FEI Company) scanning electron microscope (SEM). The operating conditions included the use of Everhart–Thornley Detector (ETD) in the secondary electrons (SE) analysis mode, high vacuum conditions and a high voltage ranging from 20 kV up to 30 kV for all measurements. For the image recording a resolution of 4096×3536 pixels was used, together with a dwell time ranging from 1 μs up to 5 μs, leading to a frame time of 11.5 seconds up to 57.5 seconds, depending on the sample and the investigated area. A multistage device was used for the measurement and the samples were fixed on the 0.5” aluminium specimen stubs using conductive carbon adhesive tape. The representative sample fragments transferred onto the SEM support was performed with minimal intervention on the particle's morphology. The samples were analyzed at room temperature.

From the three investigated samples, the least surface electrical charging under the analysis conditions was observed for sample RA6, followed by NB6 and NA6. For the NA6 sample, the image recording at high magnification conditions (>10.000×) was performed with moderate results and was only possible using a low dwell time of 1 μs. The structures of individual molecules were minimized at the quantum level using a gradient‐corrected functional, Perdew and Wang (PW91),[^33^, ^34^] including all electrons in the calculation. For these minimized structures, less or more ordered periodic structures were constructed, namely sets I and set II. The first set of structures, set I, which was intended to simulate the bulk properties of compounds in the crystalline state, was constructed from the inclusion of a single molecule in a cell, which was multiplied along the three x, y, z axes. Thus, four systems were generated, each containing 27 molecules arranged in a crystal lattice‐like structure. The Set I structures, corresponding to each compound, were minimized and then heated to room temperature (298 K) as well as to temperatures corresponding to the midpoint of the mesophase range, as were experimentally determined by DSC. This temperature, referred to as T1, corresponds to 431 K for REZ6, 390 K for NA6, and 366 K for NB6, respectively. For RA6 structure the isotropization temperature, i. e. 381 K, was considered. The Set II structures were constructed by including 10 molecules of each type in an amorphous cell, considering this time a nematic ordering. The construction of the cells was carried out starting from an initial density of 0.6 g/cm^3^, to reach a final density of 1 g/cm^3^. NVT and NPT Anneal dynamics (Forcite module) simulations were performed to find the structures with the lowest energies. For the production run, simulations were performed in the NPT ensemble for 500 ps at temperature T1, corresponding to the midpoint of the mesophase range.

### Synthetic Procedures

1‐((4‐Hexyloxyphenyl)‐4‐azophenylcarbonyloxy)‐3‐(4‐methoxyphenylcarbonyloxy)benzene (RA6): 0.24 g (1 mmol) 1‐(4‐methoxyphenylcarbonyloxy)‐3‐hydroxybenzene (2) in 30 ml CH_2_Cl_2_, 0.38 g (1.1 mmol) 4‐(4‐hexyloxyphenylazo)‐benzoyl chloride (3), 0.18 g (1.3 mmol) K_2_CO_3_, 8.4 mg (0.025 mmol) TBAHS, – orange solid, η=87 %, m.p.=109 °C.

^1^H‐NMR δH ppm (500 MHz, CDCl_3_): 8.32 (d, 2H, Ar, J=8.55 Hz), 8.15 (d, 2H, Ar, J=8.9 Hz), 7.96 (dd, 4H, Ar, J_1_=8.65 Hz, J_2_=2.35 Hz), 7.48 (t, 1H, Ar, J=8.2 Hz), 7.19 (m, 3H, Ar), 7.02 (d, 2H, Ar, J=9.0 Hz), 6.99 (d, 2H, Ar, J=8.9 Hz), 4.05 (t, 2H, −O−CH_2_−, J=6.55 Hz), 3.89 (s, 3H, −O−CH_3_), 1.83 (qv, 2H, −CH_2_−), 1.49 (qv, 2H, −CH_2_−), 1.36 (m, 4H, −CH_2_−), 0.92 (t, 3H, −CH_3_). ^13^ C‐NMR δC ppm (125 MHz, CDCl_3_): 164.63, 164.48, 164.11, 162.60, 155.96, 151.73, 151.49, 146.95, 132.47, 131.35, 130.26, 129.93, 125.44, 122.66, 121.63, 119.53, 119.14, 116.00, 114.93, 114.00 (2 *>C=O+18 C, aromatic), 68.56 (−O−CH_2_), 55.63 (−OCH_3_), 31.67, 29.23, 25.79, 22.71, 14.16 (5 C, aliphatic); m/z (CHCl_3_): 577,56 [M+2+Na]^+^. Elemental analysis calcd for C_33_H_32_N_2_O_6_ (%): C 71.72, H 5.84, N 5.07, O 17.37; found, C 72.73, H 6.61, N 6.15, O 14.51.

1‐((4‐Heptyloxyphenyl)‐4‐azophenylcarbonyloxy)‐3‐(4‐methoxyphenylcarbonyloxy)benzene (RA7): 0.24 g (1 mmol) 1‐(4‐methoxyphenylcarbonyloxy)‐3‐hydroxybenzene in 30 ml CH_2_Cl_2_, 0.39 g (1.1 mmol) 4‐(4‐heptyloxyphenylazo)‐benzoyl chloride, 0.18 g (1.3 mmol) K_2_CO_3_, 8.4 mg (0.025 mmol) TBAHS, – orange solid, η=81 %, m.p.=103 °C.

^1^H‐NMR δH ppm (500 MHz, CDCl_3_): 8.32 (d, 2H, Ar, J=8.60 Hz), 8.15 (d, 2H, Ar, J=8.9 Hz), 7.96 (dd, 4H, Ar, J_1_=8.65 Hz, J_2_=2.6 Hz), 7.48 (t, 1H, Ar, J=8.2 Hz), 7.19 (m, 3H, Ar), 7.02 (d, 2H, Ar, J=9.0 Hz), 6.99 (d, 2H, Ar, J=8.9 Hz), 4.05 (t, 2H, −O−CH_2_−, J=6.55 Hz), 3.89 (s, 3H, −O−CH_3_), 1.79 (qv, 2H, −CH_2_−), 1.46 (qv, 2H, −CH_2_−), 1.36 (qv, 2H, −CH_2_−), 1.31 (m, 4H, −CH_2_−) 0.89 (t, 3H, −CH_3_). ^13^C‐NMR δC ppm (125 MHz, CDCl_3_): 164.64, 164.48, 164.13, 162.60, 155.97, 151.73, 151.50, 146.96, 132.47, 131.36, 130.28, 129.93, 125.44, 122.66, 121.66, 119.54, 119.15, 116.01, 114.94, 114.00 (2 *>C=O+18 C, aromatic), 68.58 (−O−CH_2_), 55.68 (−OCH_3_), 31.87, 29.10, 28.84, 26.15, 22.74, 14.22 (6 C, aliphatic); m/z (CHCl_3_): 589.50 [M+Na]^+^. Elemental analysis calcd for C_34_H_34_N_2_O_6_ (%): C 72.07, H 6.05, N 4.94, O 16.94; found, C 72.91, H 6.21, N 5.89, O 14.99.

1‐((4‐Octyloxyphenyl)‐4‐azophenylcarbonyloxy)‐3‐(4‐methoxyphenylcarbonyloxy)benzene (RA8): 0.24 g (1 mmol) 1‐(4‐methoxyphenylcarbonyloxy)‐3‐hydroxybenzene in 30 ml CH_2_Cl_2_, 0.41 g (1.1 mmol) 4‐(4‐octyloxyphenylazo)‐benzoyl chloride, 0.18 g (1.3 mmol) K_2_CO_3_, 8.4 mg (0.025 mmol) TBAHS, – orange solid, η=88 %, m.p.=104 °C.

^1^H‐NMR δH ppm (500 MHz, CDCl_3_): 8.32 (d, 2H, Ar, J=8.55 Hz), 8.15 (d, 2H, Ar, J=8.9 Hz), 7.96 (dd, 4H, Ar, J_1_=8.65 Hz, J_2_=2.35 Hz), 7.48 (t, 1H, Ar, J=8.2 Hz), 7.19 (m, 3H, Ar), 7.02 (d, 2H, Ar, J=9.0 Hz), 6.99 (d, 2H, Ar, J=8.9 Hz), 4.05 (t, 2H, −O−CH_2_−, J=6.55 Hz), 3.89 (s, 3H, −O−CH_3_), 1.83 (qv, 2H, −CH_2_−), 1.48 (qv, 2H, −CH_2_−), 1.32 (m, 8H, −CH_2_−), 0.90 (t, 3H, −CH_3_). ^13^C‐NMR δC ppm (125 MHz, CDCl_3_): 164.65, 164.51, 164.13, 162.62, 155.99, 151.74, 151.51, 146.97, 132.49, 131.38, 130.28, 129.95, 125.45, 122.67, 121.66, 119.55, 119.16, 116.02, 114.95, 114.02 (2 *>C=O+18 C, aromatic), 68.59 (−O−CH_2_), 55.65 (−OCH_3_), 31.94, 29.47, 29.36, 29.29, 26.14, 22.79, 14.25 (7 C, aliphatic); m/z (CHCl_3_): 605.41 [M+2+Na]^+^. Elemental analysis calcd for C_35_H_36_N_2_O_6_ (%): C 72.09, H 6.25, N 4.82, O 16.53; found, C 72.13, H 6.54, N 5.12, O 16.21.

1‐((4‐Nonyloxyphenyl)‐4‐azophenylcarbonyloxy)‐3‐(4‐methoxyphenylcarbonyloxy)benzene (RA9): 0.24 g (1 mmol) 1‐(4‐methoxyphenylcarbonyloxy)‐3‐hydroxybenzene in 30 ml CH_2_Cl_2_, 0.42 g (1.1 mmol) 4‐(4‐nonyloxyphenylazo)‐benzoyl chloride, 0.18 g (1.3 mmol) K_2_CO_3_, 8.4 mg (0.025 mmol) TBAHS, – orange solid, η=83 %, m.p.=105 °C.

^1^H‐NMR δH ppm (500 MHz, CDCl_3_): 8.32 (d, 2H, Ar, J=8.6 Hz), 8.15 (d, 2H, Ar, J=8.95 Hz), 7.96 (dd, 4H, Ar, J_1_=8.65 Hz, J_2_=2.6 Hz), 7.48 (t, 1H, Ar, J=8.2 Hz), 7.18 (m, 3H, Ar), 7.02 (d, 2H, Ar, J=9.0 Hz), 6.99 (d, 2H, Ar, J=8.9 Hz), 4.05 (t, 2H, −O−CH_2_−, J=6.55 Hz), 3.89 (s, 3H, −O−CH_3_), 1.83 (qv, 2H, −CH_2_−), 1.49 (qv, 2H, −CH_2_−), 1.34 (qv, 2H, −CH_2_−), 1.30 (m, 8H, −CH_2_−), 0.89 (t, 3H, −CH_3_). ^13^C‐NMR δC ppm (125 MHz, CDCl_3_): 164.65, 164.51, 164.13, 162.61, 155.99, 151.74, 151.51, 146.97, 132.49, 131.37, 130.28, 129.95, 125.45, 122.67, 121.66, 119.54, 119.16, 116.02, 114.95, 114.02 (2 *>C=O+18 C, aromatic), 68.58 (−O−CH_2_), 55.65 (−OCH_3_), 32.00, 29.66, 29.51, 29.39, 29.29, 26.13, 22.81, 14.25 (8 C, aliphatic); m/z (CHCl_3_): 619.33 [M+2+Na]^+^. Elemental analysis calcd for C_36_H_38_N_2_O_6_ (%): C 72.71, H 6.44, N 4.71, O 16.14; found, C 72.49, H 6.82, N 5.15, O 15.54.

1‐((4‐Decyloxyphenyl)‐4‐azophenylcarbonyloxy)‐3‐(4‐methoxyphenylcarbonyloxy)benzene (RA10): 0.24 g (1 mmol) 1‐(4‐methoxyphenylcarbonyloxy)‐3‐hydroxybenzene in 30 ml CH_2_Cl_2_, 0.44 g (1.1 mmol) 4‐(4‐decyloxyphenylazo)‐benzoyl chloride, 0.18 g (1.3 mmol) K_2_CO_3_, 8.4 mg (0.025 mmol) TBAHS, – orange solid, η=83 %, m.p.=106 °C.

^1^H‐NMR δH ppm (500 MHz, CDCl_3_): 8.32 (d, 2H, Ar, J=8.65 Hz), 8.15 (d, 2H, Ar, J=8.95 Hz), 7.96 (dd, 4H, Ar, J_1_=8.7 Hz, J_2_=2.65 Hz), 7.48 (t, 1H, Ar, J=8.2 Hz), 7.19 (m, 3H, Ar), 7.02 (d, 2H, Ar, J=9.0 Hz), 6.99 (d, 2H, Ar, J=8.9 Hz), 4.05 (t, 2H, −O−CH_2_−, J=6.55 Hz), 3.89 (s, 3H, −O−CH_3_), 1.83 (qv, 2H, −CH_2_−), 1.48 (qv, 2H, −CH_2_−), 1.32 (m, 12H, −CH_2_−), 0.89 (t, 3H, −CH_3_). ^13^C‐NMR δC ppm (125 MHz, CDCl_3_): 164.65, 164.51, 164.13, 162.62, 155.99, 151.75, 151.51, 146.98, 132.49, 131.38, 130.28, 129.95, 125.45, 122.68, 121.66, 119.55, 119.16, 116.02, 114.95, 114.02 (2 *>C=O+18 C, aromatic), 68.59 (−O−CH_2_), 55.66 (−OCH_3_), 32.03, 29.70, 29.69, 29.51, 29.46, 29.29, 26.14, 22.82, 14.26 (9 C, aliphatic); m/z (CHCl_3_): 633.23 [M+2+Na]^+^. Elemental analysis calcd for C_37_H_40_N_2_O_6_ (%): C 73.0, H 6.62, N 4.60, O 15.77; found, C 73.93, H 6.91, N 5.23, O 13.93.

## Conflict of Interests

The authors declare no conflict of interest.

## Data Availability

The data that support the findings of this study are available from the corresponding author upon reasonable request.

## References

[1] H. K. Bisoyi , Q. Li , Chem. Rev. 2022, 122, 4887–4926. 34941251 10.1021/acs.chemrev.1c00761

[2] J. P. F. Lagerwall , G. Scalia , Curr. Appl. Phys. 2012, 12, 1387.

[3] J. Uchida , B. Soberats , M. Gupta , T. Kato , Adv. Mater. 2022, 34, 1. 10.1002/adma.202109063 35034382

[4] M. Nagaraj , Y. P. Panarin , J. K. Vij , C. Keith , C. Tschierske , Appl. Phys. Lett. 2010, 97, 5.

[5] T. Kato , J. Uchida , T. Ichikawa , T. Sakamoto , Angew. Chem. Int. Ed. 2018, 57, 4355. 10.1002/anie.201711163 29534321

[6] T. X. Ting , M. S. Sarjadi , M. L. Rahman , Crystals 2020, 10, 857.

[7] A. M. Resmerita , L. Epure , N. Hurduc , D. Adès , A. Siove , Macromol. Res. 2010, 18, 721.

[8] A.-M. Resmerita , L. Epure , S. Grama , C. Ibanescu , N. Hurduc , Open Chem. Biomed. Methods J. 2009, 2, 91.

[9] Y. Y. Xinlei Pang , L. V. Jiu-an , Lang Qin Chongyu Zhu , Adv. Mater. 2019, 31, 1904224. 10.1002/adma.201904224 31595576

[10] G. Shanker , K. R. S. Kumar , B. Paul , Liq. Cryst. 2022, 49, 1545.

[11] L. T. Thieghi , J. J. Bonvent , E. A. Oliveira , J. A. Giacometti , D. T. Balogh , Appl. Phys. A Mater. Sci. Process. 2003, 77, 911. 10.1103/PhysRevE.67.041701 12786371

[12] M. R. Karim , M. R. K. Sheikh , N. M. Salleh , R. Yahya , A. Hassan , M. A. Hoque , Mater. Chem. Phys. 2013, 140, 543.

[13] B. T. Thaker , J. B. Kanojiya , R. S. Tandel , Mol. Cryst. Liq. Cryst. 2010, 528, 120.

[14] G. R. Saad , N. H. S. Ahmed , A. A. Fahmi , M. M. Naoum , Liq. Cryst. 2018, 45, 1177.

[15] S. Kaur , H. Liu , J. Addis , C. Greco , A. Ferrarini , V. Görtz , J. W. Goodby , H. F. Gleeson , J. Mater. Chem. C Mater. 2013, 1, 6667.

[16] S. Kaur , L. Tian , H. Liu , C. Greco , A. Ferrarini , J. Seltmann , M. Lehmann , H. F. Gleeson , J. Mater. Chem. C Mater. 2013, 1, 2416.

[17] N. H. S. Ahmed , G. R. Saad , H. A. Ahmed , M. Hagar , RSC Adv. 2020, 10, 9643. 35866044 10.1039/c9ra10499b PMC9261499

[18] S. T. Ha , M. Y. Ng , R. T. Subramaniam , M. M. Ito , A. Saito , M. Watanabe , S. L. Lee , N. L. Bonde , Int. J. Phys. Sci. 2010, 5, 1256.

[19] M. Alaasar , Liq. Cryst. 2016, 43, 2208.

[20] G. Pelzl , S. Diele , W. Weissflog , Adv. Mater. 1999, 11, 707.

[21] C.-I. Ciobanu , L. Habasescu , D. Scutaru , G. Drochioiu , Lett. Org. Chem. 2015, 12, 91.

[22] T. X. Ting , M. S. Sarjadi , M. L. Rahman , Crystals 2020, 10, 1–42.

[23] S. Kumar , A. N. Gowda , Liq. Cryst. Rev. 2015, 3, 99.

[24] V. Kozmík , E. Rodinová , T. Prausová , J. Svoboda , V. Novotná , E. Gorecka , D. Pociecha , Liq. Cryst. 2018, 45, 746.

[25] S. N. F. Sardon , N. M. M. A. Rahman , M. R. Karim , N. I. Zahid , N. M. Salleh , J. Mol. Struct. 2021, 1225, 129112.

[26] C. I. Ciobanu , I. Carlescu , G. Lisa , D. Scutaru , Croat. Chem. Acta 2014, 87, 7.

[27] G. Simion , I. Carlescu , G. Lisa , D. Scutaru , Studia Univ. Babes-Bolyai Chem. 2011, LVI, 75.

[28] V. Kozmík , A. Kovářová , M. Kuchař , J. Svoboda , V. Novotná , M. Glogarová , J. Kroupa , Liq. Cryst. 2006, 33, 41.

[29] C. I. Ciobanu , G. Drochioiu , I. Carlescu , G. Lisa , V. Antoci , V. Vasilache , D. Scutaru , Lett. Org. Chem. 2016, 13, 156.

[30] S. S. Patnaik , S. J. Plimpton , R. Pachter , W. Wade Adams , Liq. Cryst. 1995, 19, 213.

[31] Beilstein Handbook of Organic Chemistry, Fourth Edition Beilsteins Handbuch der Organischen Chemie, 4. Auflage, Springer-Verlag, Berlin, 1949.

[32] G. Simion , D. F. Iuganu , I. Carlescu , D. Scutaru , Buletinul IPI 2011, LVII(LXI), 213.

[33] *Materials Studio 4.0 – Dassault Systèmes BIOVIA, Materials Studio 4.0, San Diego*, CA, USA, **2017**.

[34] J. P. Perdew , Y. Wang , Phys. Rev. B 1992, 45, 13244. 10.1103/physrevb.45.13244 10001404

